# Comparison of the effects of transdermal and oral rivastigmine on cognitive function and EEG markers in patients with Alzheimer’s disease

**DOI:** 10.3389/fnagi.2014.00179

**Published:** 2014-07-23

**Authors:** Davide V. Moretti, Giovanni B. Frisoni, Giuliano Binetti, Orazio Zanetti

**Affiliations:** Scientific Institute for Research and Care of Alzheimer’s and Psychiatric Diseases, San Giovanni Di Dio FatebenefratelliBrescia, Italy

**Keywords:** transdermal rivastigmine oral rivastigmine, Alzheimer’s disease, dementia, cognitive function, EEG

## Abstract

**Background:** Alzheimer’s disease (AD) is the most common cause of dementia in older patients. Rivastigmine (RV, Exelon, Novartis), a reversible cholinesterase inhibitor, improves clinical manifestations of AD and may enhance ACh-modulated electroencephalogram (EEG) alpha frequency. This pilot study aimed to determine the effects of two formulations of RV [transdermal patch (RV-TDP) and oral capsules (TV-CP)] on alpha frequency, in particular the posterior dominant rhythm, and cognitive function [assessed by the Mini-Mental State Examination (MMSE)] in patients with AD.

**Methods:** Subjects with AD were assigned to receive either RV-TDP 10 cm^2^ or RV-CP 12 mg/day. All patients underwent EEG recordings at the beginning and end of the 18-month study period using P3, P4, O1, and O2 electrodes, each at high (10.5–13.0 Hz) and low (8.0–10.5 Hz) frequency. MMSE scores were determined at the start of the study (T0) and at three successive 6-month intervals (T1, T2, and T3).

**Results:** RV-TDP administration (*n* = 10) maintained cognitive function as evidenced by stable MMSE scores from baseline to 18 months (21.07 ± 2.4–21.2 ± 3.1) compared with a decrease in MMSE score with RV-CP (*n* = 10) over 18 months [18.3 ± 3.6–13.6 ± 5.06 (adjusted for covariates *p* = 0.006)]. MMSE scores were significantly different between treatment groups from 6 months (*p* = 0.04). RV-TDP also increased the spectral power of alpha waves in the posterior region measured with electrode P3 in a significantly great percentage of patients than TV-CP from baseline to 18 months; 80% vs 30%, respectively [*p* = 0.025 (*χ*^2^ test)].

**Conclusions:** RV-TDP was associated with a greater proportion of patients with increased posterior region alpha wave spectral power and significantly higher cognitive function at 18 months, compared with RV-CP treatment. Our findings suggest that RV-TDP provides an effective long-term management option in patients with AD compared with oral RV-CP. This study is a pilot, open-label study with a clear explorative purpose and with a small number of patients. Further randomized, double-blind, placebo-controlled trial studies with a bigger sample size as well as healthy controls are needed to support these initial results.

## INTRODUCTION

In Alzheimer’s disease (AD), patients have reduced cerebral production of choline acetyl transferase, leading to decreased acetylcholine synthesis and impaired cortical cholinergic function (Ferri et al., 2005; Ziabreva et al., 2006; World Health Organization, 2011). Knowledge of this marked cholinergic deficit led to clinical research into the potential for therapeutically augmenting cholinergic activity (Perry et al., 1978; Caamano et al., 1994). The first agents to be investigated, acetylcholine precursors, were found to be ineffective, and postsynaptic cholinergic receptor agonists were found to have unacceptable side effects (Greenwald and Davis, 1983; Holden and Kelly, 2002). Conversely, the results of studies with acetyl cholinesterase inhibitors – agents which increase cholinergic transmission by delaying the breakdown of acetylcholine released into synaptic clefts – were encouraging (Ashford et al., 1989). To date, the use of acetylcholinesterase inhibitors is the only therapy to have shown consistently positive results in the management of AD (Holden and Kelly, 2002).

Large-scale multicenter trials have shown that rivastigmine (RV), a “pseudo-irreversible” acetyl- and butyryl-cholinesterase inhibitor with a phenylcarbamate structure, now approved for use in 60 countries, is effective in improving patients’ cognitive, behavioral, and daily functioning (Forette et al., 1999; McMillan, 1999; Rosler et al., 1999; Auriacombe et al., 2002; Burns et al., 2004; Feldman and Lane, 2007).

Rivastigmine was the first cholinesterase inhibitor to be approved as a transdermal patch (TDP) for the treatment of mild-to-moderate AD (Winblad et al., 2007). Clinical data support the RV-TDP pharmacokinetic profile (Kurz et al., 2009), demonstrating that smooth, continuous drug delivery translates into improved tolerability profile compared with the oral administration (Winblad et al., 2007; Lefevre et al., 2008). Transdermal administration also provides additional benefits including improving adherence, increased convenience and ease of use for caregivers, and a reduced impact on daily activities (Guay, 2008). Of note, a recent study has demonstrated that the Clinical Global Impressions-Change scale (CGI-C), evaluated by physicians, improved after the switch from oral to transdermal RV, suggesting a better clinical efficacy (Reñé et al., 2014).

Patients with AD are known to have abnormal cortical sources of resting-state EEG rhythms (Moretti et al., 2004). Studies also show that resting-state EEG rhythms are sensitive to early stage AD progression over a year (Moretti et al., 2004). Patients with mild AD are characterized by a power decrease in posterior alpha sources and a power increase in widespread delta sources compared with normal elderly subjects (Moretti et al., 2004). In patients with mild AD, one-year follow-up EEG recordings showed increased power of widespread delta sources and decreased power of widespread alpha and posterior beta 1 sources. This suggests that resting-state EEG sources are sensitive (at least at group level), to the extent of cognitive decline that can occur in patients with mild AD in 12 months (Babiloni et al., 2013). Resting-state EEG could be a cost-effective and non-invasive marker for AD treatment efficacy in clinical studies (Moretti et al., 2004; Babiloni et al., 2013).

The objectives of this single-center study in patients with AD were to determine the effects of two formulations of RV (transdermal and oral) on cognitive decline – as assessed by Mini-Mental State Examination (MMSE) scores. To support this effect on the cognitive decline with a neurophysiological biomarker, the EEG alpha frequency has been chosen, given the evidence indicating that changes in acetylcholine cause alterations in alpha frequency, in particular in the posterior cerebral regions alpha dominant rhythm, as it is in theses regions that the dominant frequency is most expressed (Babiloni et al., 2013).

## MATERIALS AND METHODS

### SUBJECTS

Subjects with AD were retrospectively recruited from the Translational Outpatient Memory Clinic (TOMC) of the Scientific Institute for Research and Care (IRCCS) of Alzheimer’s and Psychiatric Diseases “Centro S. Giovanni di Dio-Fatebenefratelli” in Brescia, Italy. The previous diagnosis of AD was made according to NINCDS-ADRDA criteria and the *Diagnostic and Statistical Manual of Mental Disorders IV*. Patients had been referred to the TOMC already knowing they had AD, but they were not receiving any therapy for AD prior to this study. Informed consent about the use of data for research purposes was obtained from all participants or their caregivers, according to the guideline of the local ethics committee and Code of Ethics of the World Medical Association (Declaration of Helsinki).

Patients were rated with a series of standardized diagnostic and disease severity instruments, including the MMSE (Folstein et al., 1975), the Clinical Dementia Rating Scale (CRD; Hughes et al., 1982), the Hachinski Ischemic Scale (HIS; Rosen et al., 1980), and the Instrumental and Basic Activities of Daily Living (IADL, BADL; Lawton and Brody, 1969). In addition, patients underwent diagnostic neuroimaging procedures such as magnetic resonance imaging (MRI), EEG, and laboratory testing to rule out other causes of cognitive impairment. In addition, included patients had no known problems with previous anticholinesterase therapy.

Patients with cognitive deficits, due to psychological (anxiety, depression, etc.) or physical [hypothyroidism, vitamin B_12_, and folate deficiency, uncontrolled heart disease, uncontrolled conditions (diabetes, etc.)] reasons were excluded.

This was not a study about safety or tolerability of the drug, both already previously investigated in other clinical trials. This is an explorative study that only wants to detect cognitive and neurophysiological differences in patients taking the two different forms of RV. In this view, we prefer to choose retrospectively our patients among them who have taken the higher doses in both formulations for the 18-month follow-up period. In this retrospective analysis, the higher number of patients for each group was collected. At the end of the process, 10 patients for each group were recruited. As a consequence, there was no exclusion from an initial larger number of patients.

### STUDY DESIGN

This was a single-center, clinical, explorative, non-randomized, open-label, pilot study. Subjects were assigned to receive either RV-TDP or RV capsules (RV-CP) according to patient preference or availability. Patients with RV-CP were titrated to the maximum dosage in 4-week steps over 16 weeks. In other words, those in the RV-CP group were up-titrated from 3 mg/day (1 and 5 mg, twice a day) in steps of 3 mg/day to a maximum of 12 mg/day (6 mg, twice a day).

Patients in the RV-TDP group were up-titrated from a 5 cm^2^ (4.5 mg/die) starting dose to a maximum size of 10 cm^2^ patch (9.5 mg/die) after 8 weeks. Patients were administered at the highest dose for the entire duration of the study.

The patch was applied to RV-TDP patients by caregivers to clean, dry, hairless skin on the patient’s upper back every morning and worn for 24 h, during which normal activities including bathing were allowed. To minimize possible skin irritation, patch placement on the upper back was alternated between the left and right sides, daily. RV-CP patients took a capsule with breakfast and one with their evening meal.

Given the explorative nature of the study, a power analysis was not performed to determine the study sample size.

To allow comparisons between the two groups (oral and TDP), the following EEG measurements were carried out: (1) high alpha and low alpha frequency power at each electrode separately; (2) with total high and low alpha frequency power at each electrode; (3) high alpha frequency power estimated as the sum at all four electrodes; (4) low alpha frequency power estimated as the sum at all four electrodes low; (5) total high and low alpha frequency power estimated as the sum of all four electrodes. MMSE scores were determined at the start of the study and at three successive 6-month intervals (T0, T1, T2, and T3).

### EEG RECORDINGS

All patients underwent EEG recordings at the beginning and at the end of the 18-month study period using P3, P4, O1, and O2 electrodes each at high (10.5–13.0 Hz) and low (8.0–10.5 Hz) frequency. These electrodes were chosen as the detection in alpha frequency changes is better performed on posterior brain areas. All EEG recordings were obtained in the morning with subjects resting comfortably with eyes closed. The EEG activity was recorded continuously for 5 min from 19 sites by using electrodes set in an elastic cap (Electro-Cap International, Inc.) and positioned according to the 10–20 international systems (Fp1, Fp2, F7, F3, Fz, F4, F8, T3, C3, Cz, C4, T4, T5, P3, Pz, P4, T6, O1, and O2). The ground electrode was placed in front of Fz. The left and right mastoids served as the reference for all electrodes.

Data were recorded with a 0.3–70 Hz band-pass filter and digitized at a 250 Hz sampling rate (BrainAmp, BrainProducts, Germany). Electrode skin impedance was set at <5 kHz. Vertical and horizontal eye movements were detected by recording the electro-oculogram (EOG). In order to keep a constant level of vigilance, an operator controlled the subject and the EEG traces, alerting the subject when there were signs of behavioral and/or EEG drowsiness.

The recording time of 5 min was considered optimal because, although longer recordings would have reduced data variability, they would also have increased the possibility of slowing of EEG oscillations due to reduced vigilance and arousal.

Off-line recordings were used to calibrate the scalp recordings to the common average prior to EEG artifact detection and analysis.

### ANALYSIS OF INDIVIDUAL FREQUENCY BANDS

EEG data were analyzed and fragmented off-line in consecutive 2-s epochs. A digital Fast Fourier Transform (FFT)-based power spectrum analysis (Welch technique, Hanning windowing function, no phase shift) computed the power density of EEG rhythms with a 0.5 Hz frequency resolution (range: 2–45 Hz). Two alpha frequencies – low alpha (8–10.5 Hz) and high alpha (10.5–13 Hz) – were selected according to the literature guidelines (Klimesch, 1997, 1999; Moretti et al., 2004, 2007a, b, 2008, 2009a, b).

An average of 140 epochs (range: 130–150) was analyzed. EEG epochs with artifacts underwent preliminary identification by an automatic computerized procedure (Moretti et al., 2003, 2004, 2007a, b, 2008, 2009a, b, 2011a, b), then automatic selections were double-checked and confirmed manually by two expert electro-encephalographists and epochs with muscular, ocular and other types of artifacts were discarded.

### STATISTICAL ANALYSIS

The Wilcoxon signed-rank test was performed to compare EEG spectral power measurements taken at T0 and T18. The Wilcoxon test is a non-parametric alternative to the paired Student’s *t*-test for related samples. The Chi-square test was used to identify statistical difference in non-continuous data.

A multivariate ANCOVA model was used to evaluate the effect of the MMSE baseline score on each difference between T0 and T18. To have a control analysis for the final result, difference between the MMSE score at T0 and T18 was evaluated also with an uncovaried analysis. The *p*-value was set to <0.05. For all analyses, the IBM SPSS Statistics for Windows, Version 20.0, was used.

## RESULTS

### PATIENT DISPOSITION AND BASELINE DEMOGRAPHICS AND CLINICAL CHARACTERISTICS

Twenty patients were enrolled in the study. Ten patients each were assigned to the RV-TDP and RV-CP groups. There were no significant differences in age, duration of education, or MMSE score at baseline between the two groups (**Table 1**).

**Table 1:** Socio-demographic and cognitive characteristics at baseline.

	RV-TDP	RV-CP	*p*-Value
*N* (male/female)	10 (4/6)	10 (4/6)	
Age (years)	82.2 ± 2.3	80.3 ± 2.5	0.2
Education (years)	5.4 ± 1.7	4.9 ± 2.1	0.1
MMSE	21.07 ± 2.4	18.3 ± 3.6	0.08
Disease duration (months)	38.2 ± 1.8	39.5 ± 0.8	0.2
IADL	5 lost/8	5 lost/8	

*RV-CP, Rivastigmine capsules; RV-TDP, Rivastigmine transdermal patch; MMSE, Mini-Mental State Examination; IADL, instrumental activities of daily living; (mean value ± standard deviations).*

### MMSE DATA

**FIGURE 1:**
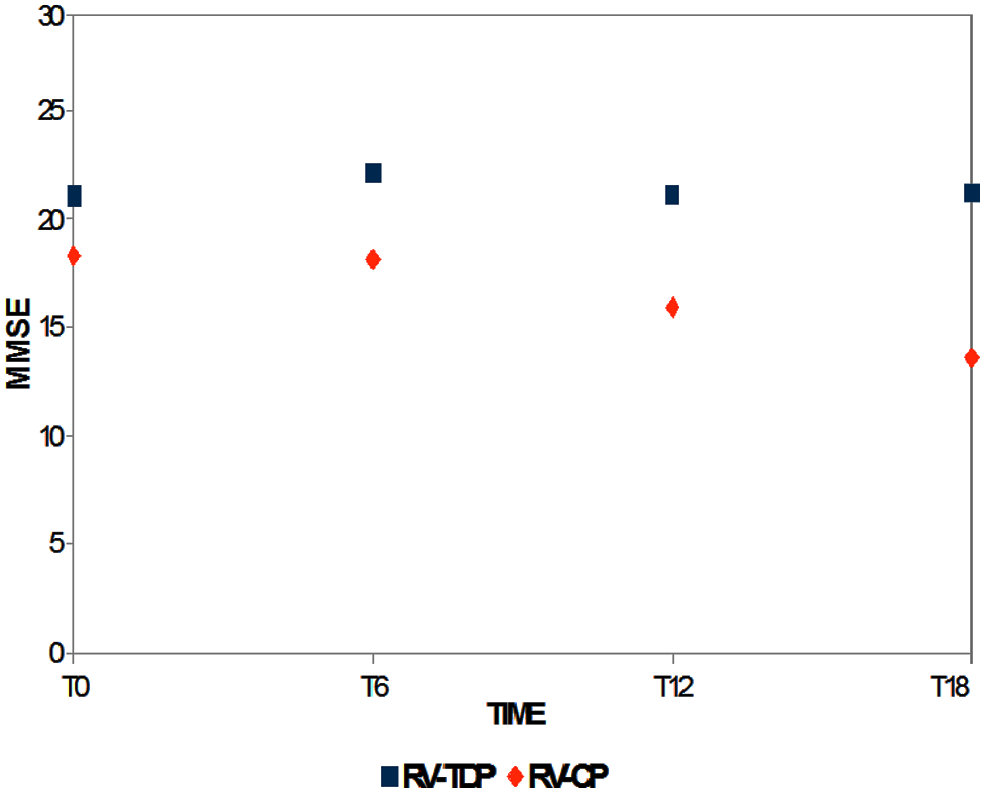
**Mean of the Mini-Mental State Examination (MMSE) score at baseline and T6, T12, and T18 follow-up in each of the two groups (RV-TDP, rivastigmine transdermal patch; RV-CP, rivastgmine capsules)**.

**FIGURE 2:**
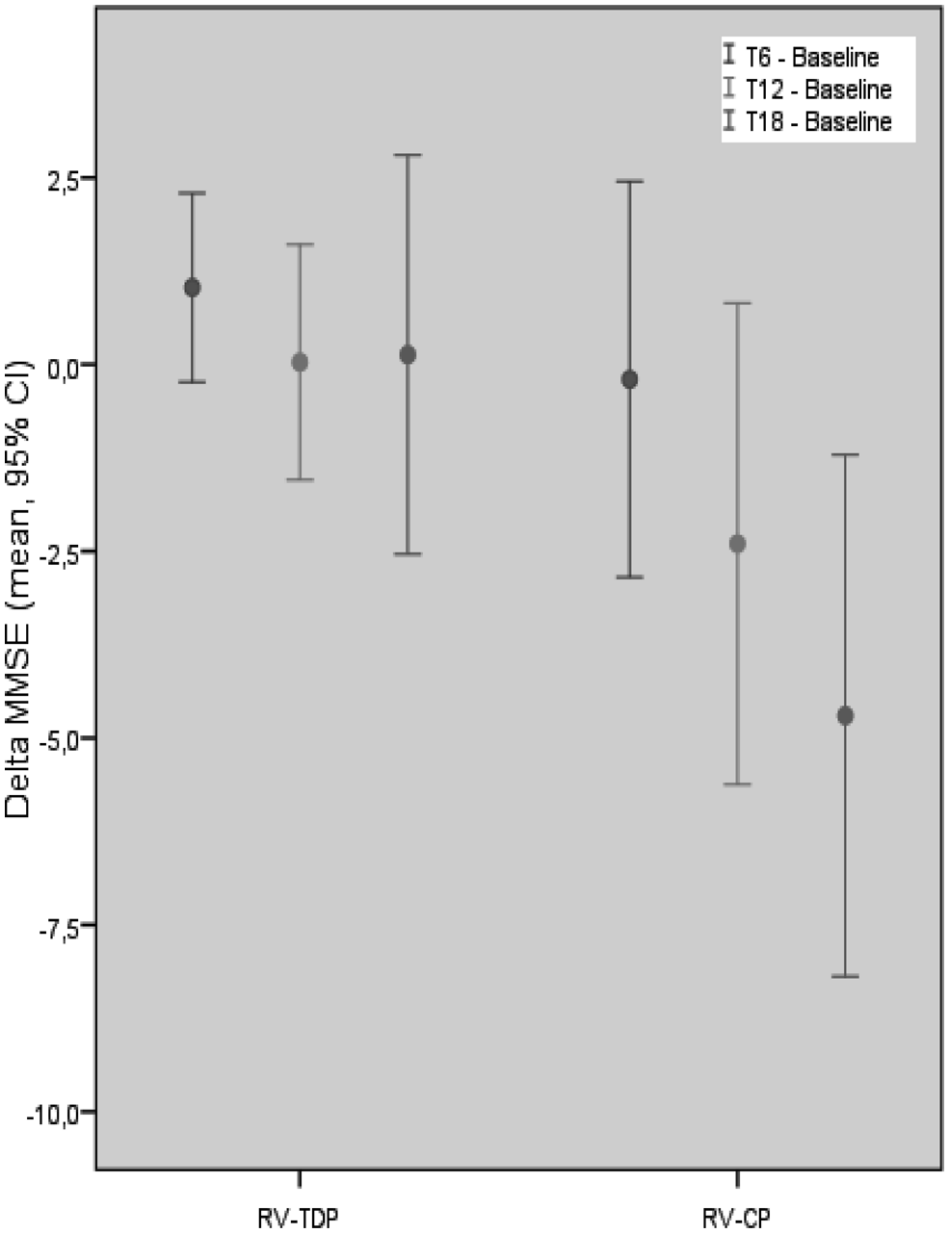
**Mean of the MMSE score difference between follow-up at T18, T12, T6, and baseline in each group with MMSE baseline score covariation (RV-TDP, rivastigmine transdermal patch; RV-CP, rivastgmine capsules)**.

Mini-Mental State Examination scores decreased in patients taking the oral formulation while remaining unchanged over the 18-month study period in those taking RV-TDP; at 6, 12, and 18 months, MMSE scores were significantly higher in patients taking RV-TDP than RV-CP (*p* = 0.04, 0.006, 0.001 at T1, T2, and T3, respectively; **Figure 1**), The one-way ANOVA showed the differences in MMSE scores at 18 months between patients in the RV-TDP and RV-CP groups were statistically significant (*p* = 0.023). Moreover, using a multivariate ANCOVA model with baseline MMSE score as a covariate, the differences between MMSE scores from baseline to 12 and 18 months were significantly greater in patients taking RV-TDP compared with those taking RV-CP (*p* = 0.042 and *p* = 0.006, for baseline to 12 months and baseline to 18 months, respectively; **Figure 2**).

### EEG DATA

**FIGURE 3:**
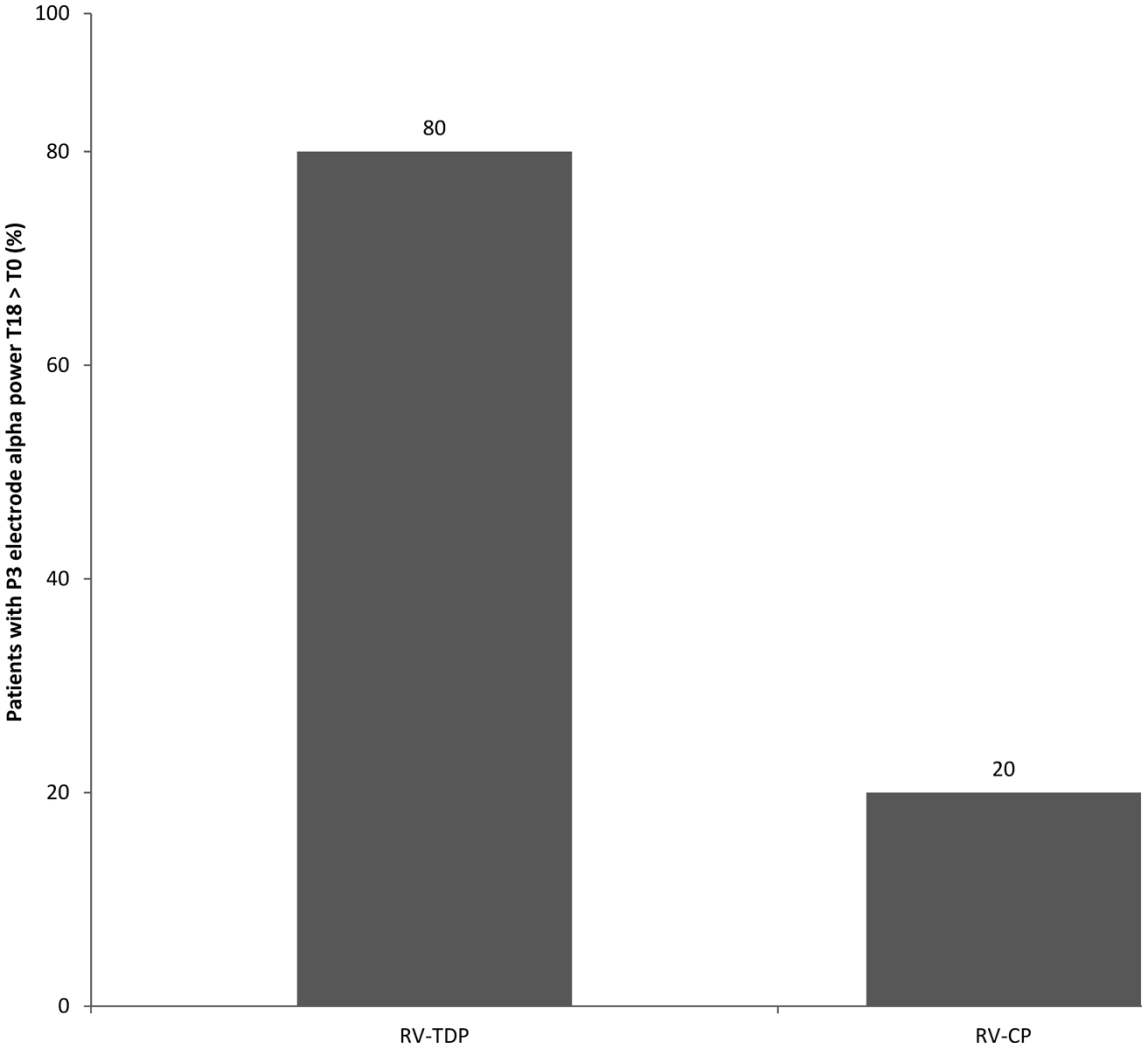
**Percentage of patients with increased alpha power at T18 follow-up as compared to the baseline measured at the P3 electrode (RV-TDP, rivastigmine transdermal patch; RV-CP, rivastgmine capsules)**.

RV-TDP and RV-CP increased spectral power (high and low alpha) in the posterior region over 18 months as measured by the sum of all the electrodes (Σ electrodes) in 8 and 4 patients, respectively, with the proportion of patients with increased spectral power at 18 months vs baseline being higher for RV-TDP than RV-CP (80 vs 40%; *p* = 0.068; **Table 2**). Applying the *χ*^2^ test, this difference reaches statistically significance for spectral power measured with electrode P3 (80 vs 30%; OR: 0.107; *p* = 0.025; **Table 2**, **Figure 3**).

**Table 2:** Wilcoxon signed-rank test results for EEG power spectra analysis.

LOW + HIGH ALPHA FREQUENCY POWER SPECTRA (8.0–13.0 Hz)				
**Electrode**	**RV**	**T18 > baseline**	**Odds ratio (95% CI)**	χ^**2**^ **test*****p*****-value**
P3	TDP	8 (80.0%)	0.107 (0.014–0.838)	**0.025**
	CP	3 (30.0%)		
P4	TDP	7 (70.0%)	0.429 (0.068–2.684)	0.361
	CP	5 (50.0%)		
O1	TDP	7 (70.0%)	0.429 (0.068–2.684)	0.361
	CP	5 (50.0%)		
O2	TDP	7 (70.0%)	0.643 (0.101–4.097)	361
	CP	6 (60.0%)		
Σ electrodes	TDP	8 (80.0%)	0.167 (0.023–1.232)	0.068
	CP	4 (40.0%)		

*RV-CP, Rivastigmine capsules; RV-TDP, Rivastigmine transdermal patch; T0, baseline visit; T18, 18-month visit; Σ, sum.*

## DISCUSSION

In this study, we compared the cognitive and neurophysiological outcomes of two groups of patients, both receiving RV, but in two different formulations – capsules andT DP. All patients were titrated to the maximum dosage, i.e., 12 mg/day for capsules and 9.5 mg/24 h for the TDP. Cognitive performance was evaluated by MMSE score, whereas neurophysiological outcomes were detected by means of alpha frequency EEG power spectra computation in the posterior regions of the brain.

Our results show that administration of RV-TDP is associated with a substantial maintenance of cognitive function as evidenced by lack of significant changes in MMSE scores in the time. Of note, MMSE scores, as well as age and education were not significantly different between the two treatment groups at baseline. Lack of cognitive impairment, evaluated by MMSE score, was evident in each of the three follow-up points (at 6, 12, and 18 months of observation) only in patients receiving RV-TDP. Moreover, the difference in the MMSE score between treatment groups was significantly different at 12 and 18 months, but not at 6 months. These results suggest that the TDP formulation stabilizes cognitive performance compared with the capsule formulation, with a significant difference seen at medium-term treatment duration, and remaining constant thereafter. Conversely, in patients taking RV-CP, there is a progressive deterioration of cognitive performance, together with a progressively greater difference compared with MMSE score in RV-TDP recipients. Our results confirm and extend previous studies showing the potential therapeutic advantage of TV-TDP vs RV-CP treatment (Winblad et al., 2007; Riedel et al., 2012). The transdermal formulation of RV is a prolonged-release drug, which avoids fluctuations in blood concentrations of RV. As a theoretical hypothesis, the constant stimulation of cholinergic receptors could have long-lasting beneficial effects on cognition over time. Moreover, maintenance of cognitive function over time could suggest a neuroprotective effect on the cholinergic system itself, preventing massive degeneration. Further studies are required to confirm this hypothesis.

It should be possible that the results have been biased by the different characteristic of the groups, with one group experiencing a more rapid decline. Even if the MMSE at baseline was lower in RV-CP group, although not significantly, it should be remarked that the composition of the groups was not based on any clinical feature, but it was completely random. Consequently, it would be more logical to think that patients with different characteristics are mixed between the two groups. In contrast, the results were positive and constant over time only in the group taking RV transdermal. Anyway, the present methodology is not suitable to address this limitation.

Regarding neurophysiological measures, our results show a major increase in the spectral alpha frequency power (both low and high alpha) in the posterior region in patients taking RV-TDP after 18 months of treatment. Previous studies have demonstrated the reliability of a pharmaco-EEG approach in evaluating both drug response and cognitive performance. Analysis of EEG-recorded electrical power (the brain field potential) is a very sensitive tool for characterization of the effects of drugs on the central nervous system (Dimpfel and Decker, 1984). Since this EEG-based method was first used, it has become increasingly clear that, depending on the particular behavioral condition or drug, the electrical power of single frequency ranges (as defined in Dimpfel et al., 1987) change independently from each other (Dimpfel et al., 1988).

The pattern of brain field potential changes with respect to specially defined frequency ranges with a specific disease or following drug application is called an “electrical activity fingerprint” (Dimpfel, 2003). This method has been used to obtain fingerprints of more than 100 compounds, including >50 standard drugs (e.g., neuroleptics, tranquilizers, analgesics, stimulants, antidepressants, narcotics, and sedatives). Interestingly, in general, these fingerprints are similar for drugs with similar indications and markedly different for drugs with different indications (Dimpfel, 2003, 2005).

A large body of literature has demonstrated that cholinergic neuromodulation augments the top–down impact of spatial attention on oscillations in the human visual cortex, specifically in the EEG alpha frequency band. Other studies have also shown that cholinergic agonists enhance the hemodynamic blood oxygenation level dependent (BOLD) response (Furey et al., 2000; Bentley et al., 2003) to attention to stimuli in visual cortex or spike-rates recorded invasively in the primary visual cortex (Herrero et al., 2008), but the studies did not examine oscillatory phenomena. Furthermore, the cholinergic impact on alpha frequency-related spatial attention effects correlated with drug-induced performance improvement (Bauer et al., 2012). This relationship indicates that the cholinergic impact on cognitive alpha frequency-related effects is not merely a secondary phenomenon.

The alpha frequency rhythm is mainly modulated by the thalamic and subcortical structure, mostly in the resting state (Steriade, 2006). An intriguing possible explanation of our results, to be verified, is that the power increase of alpha spectral power in the brain could be tuned by the peculiar modulation of butyrylcholinesterase on thalamic nuclei. The restoration of the cholinergic system in a partly physiological way, without strong peak fluctuations of drug concentrations, could prevent the synaptic loss typically seen in AD. In turn, the improved functioning of the cholinergic system is based both on the increase in alpha frequency power and the improvement of cognitive performance (Moretti et al., 2012a, b).

A range of studies have reported a strong relationship between EEG alpha activity and memory performance or intelligence, suggesting that the relationship between the dynamics of alpha frequency and cognitive performance is not correlative but causal in nature (Klimesch, 1997; Niedermeyer, 1997; Jausovec and Jausovec, 2000; Neubauer et al., 2002; Stipacek et al., 2003; Grabner et al., 2004; Doppelmayr et al., 2005b). A recent study has demonstrated that the induction of large alpha power by neuro-feedback training or repetitive transcranial magnetic stimulation (rTMS) using the alpha frequency range, exactly mimicked the typical situation for good memory performance under normal situations, thus enhancing cognitive performance (Klimesch et al., 2003). In particular, the increase in EEG alpha activity has been correlated with better retrieval of information stored in the semantic long-term memory. Moreover, in a different study, verbal-semantic and spatial-semantic performances were both associated with two different intelligence tests. The results showed that more intelligent subjects showed significantly larger alpha activity in the left hemisphere (at centro-parietal regions) compared with less intelligent subjects (Doppelmayr et al., 2005a). Our results confirm these previous studies, showing an increase in alpha power in subjects without cognitive impairment. Of note, the increase in alpha power was more evident in the left hemisphere.

An important observation is that our results show an association between the minor cognitive decline in patients taking transdermal RV and the increase in alpha frequency. This confirms previous findings that cognitive performances are best correlated with increased power in the alpha frequency. It is to remark that studies with bigger sample size will allow unambiguous correlation analysis.

Although not the focus of this study, these results add further weight to the existing evidence from our group on the possible diagnostic and prognostic role of EEG markers in patients with prodromal and diagnosed AD, an area that warrants further study.

A possible bias of the study is that changes in alpha frequency could be due to the natural course of AD. Most of the available literature shows that AD causes a decrease in alpha frequency. Our results show that this happens only in people taking RV capsules, while in patients taking RV transdermal an increase of the alpha frequency power occurs. Anyway, further studies with larger sample size and control groups are needed to conduct powerful correlation analysis.

### STUDY LIMITATIONS

This study is a pilot, open-label study with a clear explorative purpose and with a small number of patients, providing only preliminary evidence. As a consequence, it leaves the manuscript open to conjecture. However, it is an explorative study which opens future scientific pathways and debate that may be confirmed or not. It is clear that further randomized, double-blind, placebo-controlled trial studies with a bigger sample size as well as healthy controls are needed to support these initial observations and claims. The favorable outcomes observed in this study may be a useful practical guideline to help clinicians and neurologists to decide a treatment in AD.

## CONCLUSIONS

As a conclusion, findings from this study suggest that 18-month treatment with RV TDP was associated with a significantly greater proportion of patients with increased posterior region alpha wave spectral power measured using the P3 electrode, and significantly higher cognitive function as assessed using the MMSE scale, compared with oral RV.

## Conflict of Interest Statement

The authors declare that the research was conducted in the absence of any commercial or financial relationships that could be construed as a potential conflict of interest.
